# A Dictionary of Terms Employed by the French, in Anatomy, Physiology, Pathology, Practical Medicine, Surgery, Midwifery, &c. &c.; with Their Derivation from the Greek and Latin; Their Synonyms in the Greek, Latin, French, German, and English; and Illustrations in the Different Languages

**Published:** 1836-10

**Authors:** 


Art. VIII.—*A Dictionary of Terms employed by the French, in Anatomy, Physiology, Pathology, Practical Medicine, Surgery, Midwifery, &c. &c.; with their Derivation from the Greek and Latin; their Synonyms in the Greek, Latin, French, German, and English; and Illustrations in the different Languages.* By Shirley Palmer, m.d. Parts I. and II. (A.—Hui.)—*Birmingham*, 1836. 8vo. pp. 320.

This is an extraordinary book in various ways, and surely of extraordinary merit. It is founded on the well-known and very useful "*Dictionnaire des Terms de Médecine, Chirurgie, &c.*" compiled by MM. Begin, Boisseau, &c., published in Paris in 1823, and is indeed, in a considerable degree, a translation of that work, but with many additions and improvements. The most valuable and striking of the improvements is the introduction of the German and English synonymes of the greater number of the terms; but there are many others also, all calculated to render the work very superior in value and usefulness to the French Dictionary.

To gentlemen who are studious of the varied and voluminous literature of our neighbours, and particularly to those who are only commencing the study of it, this work will be truly invaluable. Whether, in making his dictionary essentially a *French* one, by selecting his alphabet and his vocabulary from that language, Dr. Palmer has consulted the interests of medical readers generally, or the utility and popularity of his work, is a different question: there can be, at least, no doubt that, for the readers of French medicine and French science, the present form of the publication is the best possible, and to all such the book itself is really indispensable.

As an illustration of the manner in which the author has executed his task, we shall extract a short passage from the second Part, just published, which will enable us to point out what we regard as defects in the general plan of the work.

"Coxal, adj.—coxalis (*coxa*, haunch or hip), L.: an epithet, in Anatomy, applied by Chaussier to the *haunch* or *hip-bone,—os coxal,—des hanches,—des iles,—iliaque,—innominé*, F.,—os coxæ,—ilii,—innominatum, L.,—hüftbein, das ungennante bein, n, G.; [composed, in early life, of three distinct pieces,—see Ilion, Ischion, Pubis; each developed by one principal and several minor points of ossification.

These pieces unite in the cotyloid cavity, to the formation of which they contribute in different proportions; and constitute, in the adult, one bone. The *os innominatum*, articulated anteriorly with its fellow bone, posteriorly with the sacrum, and with the femur below, occupies the anterior and lateral parts of the pelvis: and, if the *Gemini* are regarded as two, and the *Triceps adductor femoris* as a single muscle, each bone affords points of attachment to thirty-five muscles.]

"Coxalgie, s.f.—coxalgia, f. L. (a barbarous compound of the Latin *coxa*, haunch, with the Greek *ἄλγος*, pain),—hüftweh, n. G.,—*pain* in the *hip*: in Pathology, an affection almost invariably symptomatic of rheumatism, gout, inflammation, or organic lesion of the *hip*-joint. Coxalgique? adj.,—coxalgicus, L.,—an *coxalgie* leidend, G.,—coxalgic: suffering from *coxalgy*. By some Latin writers, it has been proposed to apply the term *Coxitis*, s.f.,—*coxite*, f.? F., to active inflammation,—hitzige entzündung des hüftgelenkes, G.,—of the hip-joint.

"Coxarthrocace, s.f. (*coxa*, the hip,—*ἄρθρον*, joint,—*κακὸς*, faulty: in Pathology, *caries* of the *coxo-femoral articulation*.

"Coxo-femoral, adj.—coxo-femoralis, L.: an epithet applied, in Anatomy, to the *articulation*,—*art. coxo-fémorale*, F.,—hüftgelenke, G.,—formed by the reception of the head of the *femoral* into the cotyloid cavity of the hip- or *coxal* bone. This articulation, designated also the *ilio-femoral*,—*ilio-fémorale*, F.,—[is maintained by a very strong capsular ligament extending from the circumference of the cotyloid cavity to the neck of the femur; by a round inter-articular ligament, which passes from the cotyloid notch to be inserted into a depression in the summit of the head of the femur; and by the fibro-cartilaginous rim,—see Cotyloïde,—which tips the margin of the acetabulum, and converts the notch into a foramen.]

"Crabe, s.m.: a genus of *Marine Crustacea*, *Cancer* (*Malacostraca*, *Decapoda*, Cuv.,—*Podophthalma*, Leach), L.,—der krebs, G.,—the Crab,—[comprising many species; the body and ten limbs of which are covered with an articulated crust, annually renewed.] Two of these species,—*C. mœnas*,—*crabe ordinaire*, F.—*καρκίνος*,—common crab, and *C. pagurus*,—*cr. tourteau* ou —*poupart*,—*πάγουρος*,—[black-clawed crab, or punger, afford a grateful, nutritious, and stimulating *aliment*, greedily sought after, but in certain conditions, either of the animal itself or of the individual feeding on it, not always taken with impunity; and quite inadmissible in congestive or inflammatory states or affections of the system.] The calcareous covering of the *latter* animal, [which, like the concretions obtained from the stomach of a species of *Astacus*, consists principally of carbonate of lime, with a minute proportion of the phosphate and gelatine,] was formerly employed in medicine, under the title of *crabs'-claws*,—*Cancri* paguri chelæ, L.,—die *krebs*scheeren, G.,—as an *antacid* and *absorbent*. All the species which now constitute the genus *Astacus*,—see E'crevisse,—were originally arranged, by naturalists, under *Cancer*." P. 172.

In the above extract all the brackets are our own, and we have inserted them to indicate what appears to us to be superfluous in a dictionary of terms, and therefore injurious, by adding unnecessarily to the matter and expense. And this we regard as a radical defect in Dr. Palmer's plan, as the same superfluity of explanation runs through the whole work. Not that we have any fault to find with the lengthened definitions and explanations, as such; as they are, for the most part, not only unexceptionable, but really superior to most others which we have met with, both in precision and conciseness; but because we regard such as altogether misplaced in a work like the present. To non-professional and unscientific persons, indeed, they may be useful, (and for these the book is not intended,) but to the whole class of professional and scientific students they must be either superfluous, as teaching what they know already, or insufficient, as attempting to convey what can be readily and more fully obtained in other works, and what must be next to useless in the scanty amount here given. The very considerable space occupied by these explanations adds greatly to the size and consequent expense of the work, and probably also is one cause of certain parts of the plan being less developed than we think is desirable. This last remark applies more particularly to the German synonyms, which are certainly omitted in many places where they ought to appear, particularly when they happen to be adjectives.

We cannot help wishing that Dr. Palmer had made his work primarily an English instead of a French dictionary; that is, had adopted the English alphabet and vocabulary in place of the French. By so doing, we think he would have produced a more useful book for his own countrymen, and, we are assured, one infinitely more popular. He could, on this plan, have introduced equally well his foreign synonyms; and, by adding to the catalogue of English vocables such French or German words as have no synonyms in English, he could have always had opportunities of giving his definition and synonym (where it existed) of every term. This defect might be supplied to the English reader by giving an alphabetical index of the *English* as well as of the *German* words; and this is absolutely necessary, to render the work useful as a book of reference to the English student. We insist the more on this point, because we see announced in the advertisement that there will be published with the last part an "Index of the *German* terms," and no mention made of an English one: the learned author must by no means omit what will be so very important—indeed so essential—an addition to his work.

The daily increasing taste for, and knowledge of, the continental languages, among the members of our profession, makes the attempt to facilitate the perfect understanding of them most praiseworthy. We are sure that the task undertaken by Dr. Palmer could not have fallen into more able hands; nor could the plan he has laid down have been better executed than in the work before us. We have, however, been long of opinion that a work on a more simple yet more comprehensive plan than any at present existing is needed in the actual state of medical literature; and we may take this opportunity of stating that the writer of this article has, in conjunction with some of his continental friends, been arranging the outlines of a plan for the construction of a work, which, it is believed, will exceed, as well in accuracy as in completeness, any thing of the same kind hitherto undertaken. We refer to a Dictionary of Medical Terms, with the synonyms in all the principal European languages, ancient and modern; the synonymy of each modern language being drawn up by a physician of the country. The work will contain no definitions beyond what are absolutely necessary for identifying the terms; and, on this account, notwithstanding the voluminous synonymy, it is hoped that it will be neither of great extent nor very expensive. Its value to medical scholars will be admitted by all. The languages intended to be comprehended in this work are the following: Greek, Latin, German, Danish, Swedish, Polish, Russian, Dutch, English, French, Italian, Spanish, and Portuguese.

We conclude by recommending Dr. Palmer's work to every student and to every practitioner who reads, or who wishes to learn, the French and German languages.

